# Feasibility of a controlled trial aiming to prevent excessive pregnancy-related weight gain in primary health care

**DOI:** 10.1186/1471-2393-8-37

**Published:** 2008-08-11

**Authors:** Tarja I Kinnunen, Minna Aittasalo, Päivikki Koponen, Katriina Ojala, Kirsi Mansikkamäki, Elisabete Weiderpass, Mikael Fogelholm, Riitta Luoto

**Affiliations:** 1 UKK Institute for Health Promotion Research, PO Box 30, 33501 Tampere, Finland; 2 Department of Health and Functional Capacity, National Public Health Institute, Helsinki, Finland; 3 Department of Medical Epidemiology and Biostatistics, Karolinska Institutet, Stockholm, Sweden; 4 Department of Etiological Research, Institute of Population-Based Cancer Research, Cancer Registry of Norway, Oslo, Norway; 5 Department of Genetical Epidemiology, Folkhalsan Research Center, Samfundet Folkhälsan, Helsinki, Finland; 6 Health Research Unit, Academy of Finland, Helsinki, Finland; 7 Tampere School of Public Health, University of Tampere, Tampere, Finland

## Abstract

**Background:**

Excessive gestational weight gain and postpartum weight retention may predispose women to long-term overweight and other health problems. Intervention studies aiming at preventing excessive pregnancy-related weight gain are needed. The feasibility of implementing such a study protocol in primary health care setting was evaluated in this pilot study.

**Methods:**

A non-randomized controlled trial was conducted in three intervention and three control maternity and child health clinics in primary health care in Finland. Altogether, 132 pregnant and 92 postpartum women and 23 public health nurses (PHN) participated in the study. The intervention consisted of individual counselling on physical activity and diet at five routine visits to a PHN and of an option for supervised group exercise until 37 weeks' gestation or ten months postpartum. The control clinics continued their usual care. The components of the feasibility evaluation were 1) recruitment and participation, 2) completion of data collection, 3) realization of the intervention and 4) the public health nurses' experiences.

**Results:**

1) The recruitment rate was slower than expected and the recruitment period had to be prolonged from the initially planned three months to six months. The average participation rate of eligible women at study enrolment was 77% and the drop-out rate 15%. 2) In total, 99% of the data on weight, physical activity and diet and 96% of the blood samples were obtained. 3) In the intervention clinics, 98% of the counselling sessions were realized, their contents and average durations were as intended, 87% of participants regularly completed the weekly records for physical activity and diet, and the average participation percentage in the group exercise sessions was 45%. 4) The PHNs regarded the extra training as a major advantage and the high additional workload as a disadvantage of the study.

**Conclusion:**

The study protocol was mostly feasible to implement, which encourages conducting large trials in comparable settings.

**Trial registration:**

Current Controlled Trials ISRCTN21512277

## Background

Obesity has become an epidemic throughout the world and increases the risk of several diseases such as type 2 diabetes, cardiovascular disease and certain cancers [1]. For women, long-term weight problems sometimes begin during pregnancy or the postpartum period [2]. A large proportion of women gain weight in excess of the recommendations [3] during pregnancy [4-6]. Excessive weight gain increases the risk of pregnancy complications, infant macrosomia, caesarean section [7] and possibly also subsequent breast cancer [8]. In addition, excessive gestational weight gain is the primary risk factor for high postpartum weight retention [4,6]. Although the average postpartum weight retention is quite small (0.5 to 3 kg) [5], up to 20 percent of women retained at least 5 kg after pregnancy in some studies [4].

The effect of dietary and physical activity habits on gestational weight gain and postpartum weight retention is still unclear [6]. Therefore, behavioural interventions are needed to study whether counselling women on diet, physical activity and healthy weight development during and after pregnancy can prevent pregnancy-related weight problems and subsequent obesity [5,6]. One exceptionally good setting for this is the Finnish maternity and child health care system, which is funded through public taxation and utilized by almost all pregnant women (99.7%) and children (98%) in Finland [9,10]. Before initiating such a large behavioural intervention, a pilot study was conducted. The aim of the present pilot study was to evaluate the feasibility of implementing such intervention in maternity clinics (MC) and child health clinics (CC) in primary health care in Finland. The feasibility was evaluated because previous interventions with similar aims [11-15] have been carried out in different settings in countries not having a primary health care system comparable to that in Finland. The specific objectives for feasibility evaluation were to assess selected indicators for recruitment and participation, completion of data collection, realization of the intervention and public health nurses' experiences on implementing the study protocol. The feasibility of the physical activity counselling has been described elsewhere in detail [16]. We hypothesized that the study protocol would be feasible to implement in the routine maternity and child health care.

## Methods

### Study design

The study was conducted in six MCs and CCs in the city of Tampere and the town of Hämeenlinna. The selection of the clinics was based on the clinics' administrative personnel's suggestion for suitable clinics. In the larger trial, a larger number of other clinics will be randomized to intervention and control clinics. The most important reason for randomizing the clinics instead of individuals – i.e. public health nurses (PHN) or pregnant and postpartum women – is the likelihood of contamination of the PHNs' counselling practices. In this pilot study, three MCs and CCs volunteered to be intervention clinics and the remaining clinics were treated as control clinics. Feasibility of the study protocol was evaluated separately in the MCs and the CCs, because the larger trial was meant to begin in early pregnancy and continue after delivery.

### Study protocol

In Finland, women with no earlier deliveries are recommended to make 11–15 visits to a PHN and three visits to a physician during pregnancy [17]. In the CCs, ten visits to a PHN and three visits to a physician are recommended during the child's first year of life [18]. The study protocol was mainly implemented on five of the routine visits to a PHN in the MC or the CC. The visits at which the women met both a PHN and a physician could not be utilized due to tight schedules.

Fourteen PHNs from the intervention clinics and nine PHNs from the control clinics participated in the study. The PHNs recruited pregnant and postpartum women with no previous deliveries for the study. The exclusion criteria were age under 18 years, type 1 or 2 diabetes mellitus, twin pregnancy, physical disability preventing exercise, substance abuse, treatment or clinical history of any psychiatric illness, otherwise problematic pregnancy (defined by a doctor), inadequate language skills in Finnish and intention to change residence within three months. The PHNs recruited the pregnant women by phone when making an appointment for the first MC visit. The postpartum women were recruited when the PHN visited the participant's home after delivery or on the participant's first visit to the CC. As this was a pilot study, the sample size was not based on power calculations. Instead, the aim was to recruit at least 40 pregnant and 40 postpartum women from the intervention and the control clinics (160 participants in total). All participants provided written informed consent to participation. The study was approved by the Ethics Committee of the Pirkanmaa Hospital District.

Data collection was similar in the intervention and the control clinics (Table 1). The PHNs sent the baseline questionnaire to the participants' homes and the participants returned the completed questionnaire on their first visit. The follow-up questionnaires and the first feedback questionnaire were completed in the waiting room before the consultation. The researchers sent the last feedback questionnaire to the participants' homes and the participants returned them in sealed envelopes on their last visits. The PHNs took a copy of the standard maternity cards of the pregnant and the postpartum participants after delivery. As an additional measurement not generally included in care, they also measured the weight and waist circumference of the postpartum women at each of the five visits. The PHNs used a structured form to record the incidence of selected adverse events reported by the participants at four of the visits. These adverse events included vaginal bleeding, strong contractions (in pregnant participants), dizziness, dyspnea, headache, chest pain, excessive tiredness or fatigue experienced by the participant, calf pain or swelling and musculoskeletal symptoms. Blood and nipple aspirate fluid (NAF) samples were collected in order to measure the selected hormones and growth factors related to breast cancer risk. To obtain a milk-free NAF sample, the NAF samples were collected at least one month after the participant had stopped breastfeeding. The blood and NAF samples were taken by the Medical Laboratory Technologists of the UKK Institute. No attempt was made to collect data from the drop-outs.

**Table 1:** Data collection in the intervention and the control clinics

	**Pregnant women ** (weeks' gestation)	**Postpartum women ** (months postpartum)
*Questionnaires for the participants*		
Background, physical activity, diet, psychosocial wellbeing	8–9	2
Physical activity, feedback for physical activity counselling	16–18	5
Diet, feedback for dietary counselling	22–24	5
Background, physical activity, diet, psychosocial wellbeing	36–37	10
Feedback for the physical activity and dietary counselling and the study as a whole	36–37	10
*Other data collection*		
Adverse events form	16–18, 22–24, 32–34 and 36–37	3, 5, 6 and 10
A copy of the maternity card (weight development and other data on pregnancy)	after delivery	2
Weight and waist circumference measurement form	-	2, 3, 5, 6 and 10
Blood sample	9–11 and 36–37	2–2.5 and 8
A 3-day food record prior to the blood sample	9–11 and 36–37	-
Nipple aspirate fluid (NAF) sample	-	8–12

**Figure 1:**
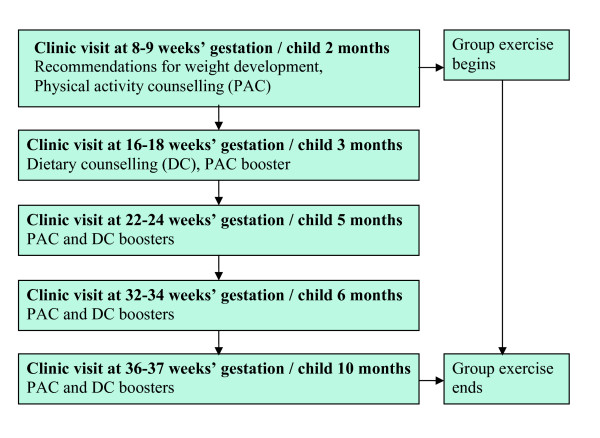
Intervention in the intervention clinics.

In the control clinics, the PHNs continued their usual physical activity and dietary counselling practices. In the intervention clinics, the intervention included individual counselling on physical activity and diet and an option to attend supervised group exercise sessions once a week at a location close to the clinic (Figure 1). The content of the intervention is described in more detail elsewhere [19,20]. Briefly, the purpose was to promote leisure time physical activity and healthy dietary habits and thereby to hold the gestational weight gain of the pregnant participants within the recommended range [3] and to support the postpartum participants' return to their pre-pregnancy weight during the study. The PHNs proceeded in the counselling with the help of counselling cards, and 20–30 min was allocated for each primary counselling session and 10–15 min for each booster session. The participants used a follow-up note book to keep record of their compliance with the physical activity and the dietary plans agreed at the counselling sessions.

Before the study began, the PHNs of the intervention clinics were trained in the counselling procedures and the study arrangements (12 h in total) and the PHNs of the control clinics for the study arrangements (6 h in total) by the research group. All PHNs received a handbook in which the tasks related to each visit were explained. One or two researchers visited each clinic monthly during the study. One supportive meeting was held for the PHNs of the intervention and the control clinics separately. The exercise instructors (n = 10) were trained for the group exercise sessions by the research group (10 h in total).

### Baseline comparability of the clinics

The location of the clinics should not have any effect on the services the clinics provide, because all clinics are supposed to follow the national guidelines for maternity and child health care [17,18]. In these guidelines, the number, timing and main content of the visits are defined. On the other hand, PHNs' work is quite autonomous and they implement counselling in their personal way.

Information on the background characteristics and the usual counselling practices of the PHNs was collected by a questionnaire (n = 21) before the PHNs were trained for the study. The responses varied between the six MCs and CCs, but the numbers of PHNs in each clinic were too small to test the statistical significance of the between-clinic differences. Concerning all clinics, 15 (71%) PHNs were aged 40 years or more. The PHNs had either the official degree of PHN (n = 15), midwife (n = 1) or both (n = 5). Of those PHNs who had worked in a MC, the median time of working in a MC was 3.5 (range 1 to 30) years. Likewise, of those PHNs who had worked in a CC, the median time of working in a CC was 13.0 (range 1 to 26) years. The counselling practices varied remarkably between the PHNs, but not between the PHNs of the intervention and the control clinics [19,20].

In Finland, the clinic attended by each pregnant and postpartum woman is determined by her place of residence. The socioeconomic background of the residents varies between these areas, which may also have affected the characteristics of the participants. There was some variation in the participants' mean age, mean pre-pregnancy BMI, education level, smoking status and the baseline dietary and physical activity habits between the six clinics (results not shown), but the statistical significance of the differences could not be tested due to the small number of participants in each clinic. Information on these variables has been reported earlier in the intervention and the control clinics [19,20].

### Feasibility evaluation

The feasibility assessment of the study protocol comprises the following four components. The main indicators and data collection for each component are described below.

#### 1) Recruitment and participation

Information on the achievement of the recruitment aim (40 participants per group, 160 in total) within three months, the participation rate of the eligible women and the drop-out rate of participants were obtained from the standardized recruitment forms used by each PHN.

#### 2) Completion of data collection

The proportion of data obtained on weight development, diet and physical activity was assessed from the number of completed and returned baseline and follow-up questionnaires, maternity cards and postpartum weight measurement forms. Information on the proportion of blood samples obtained was collected from the laboratory records.

#### 3) Realization of the intervention

Concerning the intervention clinics, the realization rate, content and duration of counselling sessions was assessed from the PHNs' counselling cards. Each counselling session was regarded to have been implemented as intended if all essential parts of the counselling card were filled in for the session. The proportion of women completing ≥ 75% of the weekly records for physical activity and diet was obtained from the participants' follow-up notebooks. The mean participation percentage in the group exercise sessions was determined by calculating first the participation percentage of each woman separately from the number of sessions available for her and then averaging the individual participation percentages. Information on participation was obtained from the participant lists kept by the exercise instructors.

#### 4) The PHNs' experiences

Information on the PHNs' opinions of the appropriateness of the training for the study was collected using a questionnaire three months after the initiation of the study. The PHNs assessed the training on the Osgood scale (1 = very poor ... 5 = excellent). Additionally, the advantages and the disadvantages of the study for the PHNs were inquired from them by a semi-structured interview within two weeks each PHN's last participant had finished the study.

## Results

### Recruitment and participation

**Figure 2:**
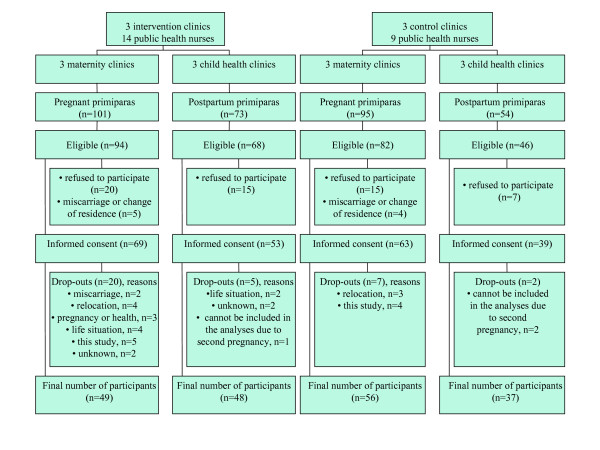
Participant flow.

As only 113 participants were enrolled within the three months allocated, the recruitment period was prolonged to six months (Table 2). Finally, of all potential 327 women with no earlier deliveries, 277 women were eligible and 224 women participated in the study (Figure 2). The participation rate was lower in the intervention than in the control clinics (Table 2). The drop-out rate was low (≤ 11%) in all clinics except for the intervention MCs (29%). Figure 2 presents the reasons for dropping out. Of the reasons related to this study, some drop-outs in the intervention clinics were unwilling to fill in more questionnaires, food records or the follow-up notebook. Other reasons, reported mostly in the control clinics, were reluctance to give the blood samples or difficulties to find the time for this.

**Table 2:** Feasibility of the study protocol^1^

**Components and main indicators**	**Maternity clinics**		**Child health clinics**		
	**Intervention**	**Control**	**Intervention**	**Control**	**Total**
**1) Recruitment and participation**					
Aim for recruitment achieved within three months (40 of participants per group, 160 in total)	yes	no	no	no	no
Participation rate of eligible women (%)	73	77	78	85	77
Drop-out rate of participants (%)	29	11	9	5	15
					
**2) Completion of data collection**					
Proportion of data obtained on weight, physical activity and diet (%)	96	100	99	100	99
Proportion of blood samples obtained (%)	98	95	96	97	96
					
**3) Realization of the intervention**					
Realization rate of counselling sessions (%)^2^	98		98		98
Duration (min) of counselling sessions, mean (sd)					All mean durations were within allocated time^2^
Primary sessions	24.0 (4.7)		25.9 (8.3)		
Booster sessions	10.4 (3.6)		10.5 (3.3)		
Proportion of women completing ≥ 75% of the weekly records for both physical activity and diet (%)^3^	87		85		86
Participation percentage in group exercise sessions (mean, sd)	38.6 (28.3)		50.7 (28.5)		45.1 (28.7)

^1 ^The drop-outs are included only when evaluating the component 1). The results concerning the component 4) are described in the text only.

^2 ^None of the counselling cards was missing.

^3 ^Numbers of missing follow-up note books were 3 in the maternity clinics and 1 in the child health clinics.

### Completion of data collection

The proportions of data obtained on weight, physical activity and diet were 96–100% and the proportions of blood samples obtained were 95–98% (Table 2). The blood samples were obtained as intended at 11.5 (sd 2.1) and 36.5 (sd 0.7) weeks' gestation or at 2.6 (sd 0.3) and 8.3 (sd 0.3) months postpartum on average. The food records were obtained from all pregnant participants except for one at baseline and two at follow-up. Of the postpartum participants, the NAF samples were obtained from 41 (48%) participants, on average at 12.1 (sd 3.3) months postpartum. NAF samples were not obtained from participants who had not stopped breastfeeding at least one month ago (n = 11), participants who were not willing to give the sample (n = 9), changed residence (n = 2) or for unknown reasons (n = 6). In addition, the Medical Laboratory Technologists did not succeed in collecting sufficient NAF from 16 women. All but two adverse events forms were returned.

### Realization of the intervention

In the intervention clinics, 98% of the counselling sessions were realized as intended and the mean durations of the sessions were as intended (Table 2). All primary counselling sessions were realized. The proportion of participants completing the follow-up notebook records regularly (≥ 75% of weekly records) was high (86%). The mean participation percentage in the group exercise sessions was higher among the postpartum than the pregnant women (51% vs. 39%).

### Public health nurses' experiences

Using the 5-point scale, the PHNs of the intervention clinics scored the training for study arrangements 3.4 (sd 1.2), physical activity counselling 3.9 (sd 1.1) and dietary counselling 3.6 (sd 1.1) on average. The PHNs of the control clinics scored the training for study arrangements 3.9 (sd 0.7) on average. Nearly all PHNs regarded the training and the support during the study as sufficient and the researchers' visits to the clinics as useful.

The PHNs of the intervention clinics considered the increased knowledge on physical activity and diet and the improved counselling skills to be the major advantages of the study for them. The PHNs of the control clinics appreciated the training they were promised after the study. The major disadvantage reported by the PHNs was that implementation of the study protocol took too much time. The extra time needed for the visits was 40–60 min/visit in the intervention clinics and 10–20 min/visit in the control clinics on average.

## Discussion

We evaluated whether a study protocol aiming at preventing excessive gestational weight gain and postpartum weight retention could be feasibly implemented in the Finnish maternity and child health care system. Integrating a study protocol into the routine functions of primary health care is a demanding task, but we managed to implement the protocol mostly as intended.

The overall participation rate was high (77%) and the drop-out rate low (15%). Data on weight development, diet and physical activity was collected very successfully. The proportion of blood samples obtained was extremely high, indicating that collection of this kind of material is possible in studies conducted in real health care settings. In the intervention clinics, almost all counselling sessions were realized as intended and most participants recorded their adherence to the physical activity and dietary plans regularly to their follow-up notebook. This success reflects the PHNs' and the participants' strong commitment to the study, possibly because they were able to see the importance of the study and to see some personal benefits compensating the burden.

Although the main experiences were positive, some problems were encountered. The recruitment time needed to be prolonged because the recruitment of the participants was slower than expected. The experience helps in estimating the realistic time needed for recruitment in further studies. The participation rate was slightly lower in the intervention clinics than in the control clinics, which may be related to the participants' background characteristics, to their reluctance to improve or monitor their dietary and physical activity habits or to the PHNs' motivation to recruit participants. In further studies, the way in which study is introduced to the participants and how they are motivated to participate in it will be especially important.

The drop-out rate was higher in the intervention MCs than in the other clinics, but the reasons for drop-out seemed quite plausible in all clinics. Pregnancy is often associated with changing residence and, consequently, clinics. For some women who changed residence, we managed to collect the follow-up questionnaires and the maternity cards by mail, thus preventing them dropping out of the study. Some women withdrew for reasons related their pregnancy (such as twin pregnancy or risk for premature delivery) or a stressful life situation. Additionally, as postpartum weight retention was the main outcome for postpartum participants in the effectiveness analyses, we had to exclude women who were pregnant again 10 months after their first delivery. On the other hand, the drop-outs related to the missing blood samples actually occurred due to misunderstanding because the participants would have been allowed to continue the study despite not giving the blood samples. Some participants in the intervention clinics withdrew because they found the data collection too burdensome. Therefore, the amount of data collection should be paid more attention in further studies. For this pilot study, we collected feasibility information, which may not be necessary in further studies.

Although the other data collection was successful, NAF samples were obtained from only 41% of the postpartum participants. The sample could not be obtained from women who were still breastfeeding when the collection of the samples was finished. Further studies should allocate a longer time period for collection of NAF samples. As NAF samples are not routinely collected in health care, some women may have been suspicious or afraid of giving them. To minimize the number of women refusing to give the sample, the methods of collecting NAF sample should be described in detail to the participants beforehand. However, the most frequent reason for a missing NAF sample was that no NAF could be obtained from the breast despite attempts. Other studies have also reported difficulties in obtaining NAF [21]. Therefore, collection of NAF samples can also be expected to be laborious in future studies.

The average participation percentage in the group exercise sessions was relatively low, especially among the pregnant participants. No information is available on the reasons why the women did not participate more often. The reasons have been discussed earlier [16] and they may be related to the fact that these pregnant women with no previous deliveries were physically relatively active before pregnancy. Therefore, part of them may have preferred to continue their previous physical activity habits instead of participating in these group exercise sessions. In further studies, one option could be to arrange group exercise sessions less often for pregnant participants. Additionally, the exercise instructor could keep in touch with the participants between the sessions to encourage them to adhere to their individual physically activity plans.

The quality and adequacy of training of the PHNs will be of crucial importance in future studies, since the PHNs regarded the increased knowledge about physical activity and diet as well as improved counselling skills as the major advantage of participating in the study. However, as the PHNs found that the implementation of the study protocol was time-consuming, the time spent on study arrangements and all paper flow should be kept to a minimum in further studies. A risk group approach should also be considered to limit the PHNs' workload and to better target counselling at those in need. Allocating shorter times for counselling may impair the effectiveness of the intervention.

A major strength of the study was that the counselling was implemented during routine visits to primary health care instead of using extra study personnel. Using this approach, we also aimed at developing counselling practices, which could be incorporated into real health care situations. Safety issues are especially important when implementing interventions among pregnant and postpartum women. Therefore, another major strength of the study was that no statistically significant differences were observed in the incidence of selected adverse events between the intervention and the control groups [16]. Nor were any between-group differences observed among pregnant participants in pregnancy or foetal outcomes [19].

One limitation of the pilot study was that we were not able to randomize the clinics, which may have caused some baseline differences between the participants of the intervention and the control clinics [19,20]. However, the participants could not choose their clinic, which may have decreased this bias. In any case, randomization of the clinics is a priority, which should be highlighted when recruiting the clinics for further studies.

## Conclusion

Implementation of the study protocol proved to be feasible in this setting, which encourages the undertaking of a large study in Finland and possibly also in other countries with maternity and child health care services funded by public taxation [22]. Such behavioural intervention studies are needed to develop maternal health care services in order to prevent pregnancy-related weight problems and the associated health problems relevant to public health.

## Competing interests

The authors declare that they have no competing interests.

## Authors' contributions

TIK participated in the development of the study design and the intervention protocols (especially dietary counselling) and in the acquisition and analysis of the data. MA participated in the development of the study design and the intervention protocols (especially physical activity counselling) and in the acquisition of data. PK and EW were involved in the development of the study design. KO participated in the development of the intervention protocols (group exercise sessions) and in the acquisition of the data. KM participated in the development of the intervention protocols (laboratory issues) and in the acquisition of the data. MF was involved in the development of the study design and the intervention protocols. RL was the principal researcher and responsible for the study concept and design and she participated in the development of the intervention protocols. All authors participated in the interpretation of the data and preparation of the manuscript. Additionally, all authors have read and approved the final manuscript.

## Pre-publication history

The pre-publication history for this paper can be accessed here:


